# Unraveling the Progression of Colon Cancer Pathogenesis Through Epigenetic Alterations and Genetic Pathways

**DOI:** 10.7759/cureus.59503

**Published:** 2024-05-02

**Authors:** Asal Abolghasemi Fard, Afshin Mahmoodzadeh

**Affiliations:** 1 Department of Cellular and Molecular Biology, Faculty of Modern Science and Technologies, Tehran Medical Sciences, Islamic Azad University, Tehran, IRN; 2 Department of Biology, Roudehen Branch, Islamic Azad University, Tehran, IRN

**Keywords:** de novo methylation, histone modification, dna methylation, epigenetic modifications, colorectal cancer

## Abstract

In the modern age, colon cancer has attained a widespread status, affecting a considerable number of people. It develops due to the progressive accumulation of genetic and epigenetic alterations. While genetic mutations have been extensively studied in the context of colon cancer, emerging evidence highlights the pivotal role of epigenetic alterations in its pathogenesis. These alterations ultimately result in the transformation of normal colonic epithelium into colon adenocarcinoma. Key mechanisms of epigenetic modifications include DNA methylation, histone modification, and nucleosome positioning. Research findings have linked these modifications to the development, progression, or metastasis of tumors. Through the assessment of the colon cancer *epigenome*, it has been discovered that practically all colorectal cancers (CRCs) display gene methylation abnormalities and changes in miRNA expression. Advancements in this area indicate that epigenetic modifications will likely be commonly used in the near future to direct the prevention and treatment of CRC. The maintenance of genome stability is essential for preserving cellular integrity. The development of CRC is primarily influenced by the loss of genomic stability, which allows for the emergence of new mutations contributing to tumor characteristics. Although genetic mutations have been extensively researched in the realm of colon cancer, recent evidence underscores the pivotal role of epigenetic changes in its pathogenesis. The following types of genomic instability will be discussed: chromosomal instability, microsatellite instability, CpG island methylation phenotype, and aberrant DNA methylation.

## Introduction and background

It has been recognized that tumors are defined by the presence of genomic alterations. Colorectal cancer (CRC) is a widespread disease that occurs as a consequence of the progressive accumulation of genetic and epigenetic alterations, ultimately causing the transformation of normal colonic epithelium into colon adenocarcinoma [1,2]. In 2022, approximately 10% of estimated cancer deaths in the United States among males and females were due to colon cancer, making it the second leading cause of cancer-related mortality globally [2]. Mutations in genes have been widely acknowledged as crucial in the formation of cancer. Conversely, alterations in epigenetics have only been identified as substantial factors in cancer progression in recent times [3,4,5]. Developmental biologist Conrad H. Waddington, in 1942, was the first person to come up with the term *epigenetics* from the combination of two terms epigenesis and genetics [5]. The epigenetic phenomenon involves reversible hereditary changes in gene expression and function that can be inherited by the next generation of cells through meiosis and mitosis, without any alterations in the DNA sequence, ultimately leading to tumorigenesis. Known as a major hallmark of human cancers, epigenetic alterations play a key role in silencing tumor suppressor genes (TSGs), activating oncogenes, thereby altering multiple cellular processes [5]. Aberrant DNA methylation patterns, abnormal histone modifications, and modified expression levels of non-coding RNAs, such as miRNAs, are commonly observed epigenetic changes in cancer. DNA methylation, being the predominant epigenetic process, significantly influences the regulation of gene expression. Epigenetic changes contribute to the pathology and molecular variety of these malignancies. This is demonstrated by the identification of a subtype of CRC known as the CpG island methylator phenotype (CIMP), which has a distinct epigenome and an elevated prevalence of methylated genes [6]. Hyper-methylation and hypo-methylation of the CpG region suppress the transcription of cancer-suppressing genes and shut down these genes. This affects all types of human cancers such as CRC [6,7]. Understanding the molecular basis is crucial as it allows for the identification of the factors responsible for initiating development, maintaining progression, and influencing the response to or resistance against antitumor agents. Within the following overview, we will briefly address the basic principles of epigenetic modifications in CRC, including DNA methylation and histone modifications, and then discuss the genetic regulation of CRC development [7].

## Review

Colorectal cancer

Recent advances in biological agent-based therapies show that we have faced dramatically increasing survival rates of CRC patients because, in societies with observation programs, the frequency rate, as well as the mortality rate, has descended since the prior stages at which the tumors are identified [8,9,10]. CRC is responsible for the majority of cancer-related deaths in the Western world, making it the foremost cause of mortality in this region [11]. The frequency rates of colon cancer are high in North America and northern Europe, lower in southern Europe, and much lower in Asia and Africa [11]. CRC progression is thought to be a multistep process that begins with polyps on the epithelial lining, progresses to benign adenomatous polyps, then to pre-malignant or malignant invasive carcinoma, and finally metastasis [11]. CRC develops slowly over a long time. About 5% to 10% of CRCs have been estimated to have significant hereditary factors. While many acquired activating factors remain unknown, approximately 3% of CRCs have been linked to common genetic syndromes, such as Lynch syndrome, Gardner syndrome, and familial adenomatous polyposis (FAP) [12]. In addition, various factors, including diet, lifestyle, environmental parameters, epigenetics, and food-borne mutagens, play a significant role in the occurrence of CRC [13,14].

Epigenetics in colon cancer

**Figure 1:**
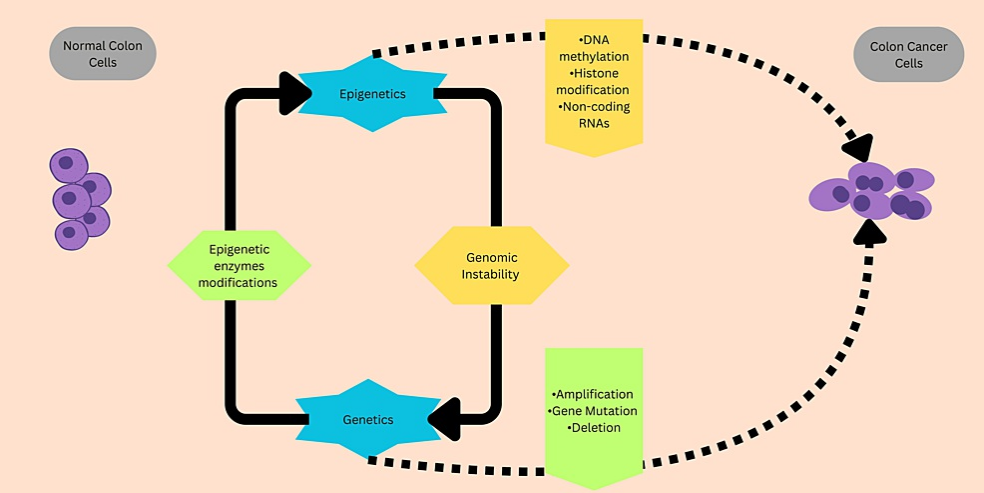
Genetic and epigenetic alterations in colorectal cancer. Genetic and epigenetic alterations are linked with each other and can lead to cancer development and progression. Original image depicted. Image credit: Asal Abolghasemi Fard.

Recent investigations have indicated that, in addition to traditional genetic modifications, epigenetics has an extensive role in carcinogenesis. In 1942, Conrad H. Waddington, a biologist, was the primary individual who came up with the term *epigenetics*. Epigenesis is an old phrase that is used to describe the differentiation of cells from their initial totipotency state during embryonic development. It incorporates reversible hereditary changes in gene expression and function that can be passed on to the next generation of cells through meiosis and mitosis without any alterations in the DNA sequence. Epigenetic modifications are consistently linked to various enzymes and cellular components and are also susceptible to external influences and can be reversed. Therefore, it is reasonable that aberrant epigenetic changes may result in improper gene expressions and contribute to the development of tumors [14]. Epigenetic abnormalities are prevalent in most CRCs. In carcinoma, the epigenetic modifications involve DNA methylation (hypermethylation and hypomethylation), modifications of histones, positioning of nucleosomes, and the regulation of non-coding RNAs, particularly in micro-RNA (small, highly conserved non-coding RNA molecules involved in the regulation of gene expression) expression. Additionally, these abnormalities coincide with classical genetic changes such as P53 [15], K-ras, and b-catenin mutations [16]. The complement of these modifications, together called the epigenome, has been involved in numerous cancers. They collaborate to guide the genome's function through modifications to neighboring basic chromatin elements [17]. The genome's compact architecture plays an important role in gene activation and repression [17,18] (Figure 1).

 DNA methylation

Three decades ago, Feinberg and Vogelstein made a significant discovery regarding the primary epigenetic alteration in cancer. They observed a substantial decrease in 5'-methylcytosine levels in CRC compared to the normal colon. This finding sheds light on the importance of DNA methylation, which is one of the most extensively studied epigenetic modifications in mammals. DNA methylation serves as a reliable mechanism for gene silencing, such as on the inactive X chromosome, and plays a crucial role in regulating gene expression. It works in relation to histone modifications and other chromatin-related proteins to facilitate these regulatory processes [19,20]. The regular control of gene activity during development heavily relies on DNA methylation, and any defects in this process can result in significant phenotypic consequences. In mammals, methylation is mainly limited to CpG dinucleotides, which are generally scarce throughout the genome, except for specific regions referred to as CpG islands, commonly found in promoters [21]. Additionally, methylation can also occur in regions containing repetitive sequences like centromeric repeats, retrotransposon elements, and ribosomal DNA (rDNA). Around half of the protein-coding genes in the human genome contain CpG islands. These regions, rich in CG content, are predominantly situated at the 5′ end of human gene promoters, displaying a 60% methylation pattern [22,23] and being present in 60% of human gene promoters. CpG islands are characterized by their length exceeding 200 base pairs, with over 50% G+C content and CpG frequency, at least 0.6 of which is statistically predicted [24]. It has been estimated that there are approximately 29,000 CpG islands in the human genome [24,25], with about one-third of all diseases caused by familial mutations and single-nucleotide polymorphisms being identified in methylated CpG sites [26]. In the normal genome, this epigenetic event occurs universally, and in human cells, 70%-80% of all CpG dinucleotides have high levels of methylation [26,27].

A recently uncovered pathway, known as CIMP, has been identified as a contributor to certain types of cancers, where gene silencing and epigenetic changes occur simultaneously due to DNA methylation [27]. CIMP is a recently discovered mechanism for carcinogenesis in colorectal carcinoma and other carcinomas like gastric, melanoma, breast, prostate, bladder, hepatocellular, and endometrial cancer [27,28], which is characterized by the methylation of multiple CpG islands. It is hypothesized that the methylation of multiple CpG islands may be due to either aberrant de novo methylation caused by a mutation in DNA methyltransferase (DNMT), or the absence of protection against de novo methylation due to the loss of a trans-activating factor [29]. Cancers with the CIMP exhibit aberrant DNA methylation, leading to concordant promoter hypermethylation of multiple genes [30]. While CIMP- cancers develop alongside a more traditional genetic instability pathway, with an excessive rate of p53 mutations and chromosomal modifications, CIMP+ cancers evolve with errors in sporadic mismatch via hypermethylation of the hMLH1 promoter, p16, and THBS1. Additionally, CIMP+ cancers represent the majority of tumors with K-ras mutations through an unknown mechanism [30].

Methylation of CpG island promoters and non-promoter CpG islands

The evidence concerning CpG island methylation in CRC has revealed some patterns that enhance the genetic investigation of cancer [30]. Research has shown that CpG island promoters in mammals remain unmethylated in normal cells. Nonetheless, during development, certain CpG island promoters undergo methylation, leading to persistent transcriptional suppression such as imprinted genes or X-chromosome inactivation [30,31]. CpG islands suggest that methylation of cytosine can hinder the binding of transcription factors (TF binding), but repression primarily occurs indirectly through the activation of chromatin changes by methyl-CpG binding domain (MBD) proteins. The degree of repression may depend on the proximity of CpGs within the promoter [32]. Methylated cytosines are prone to hyper-mutation through unconstrained deamination, resulting in the formation of uracil bases and potentially slower repair processes. The human genome has fewer CpG sites as a result of this evolutionary process; in fact, CpGs are only found at 20% of the statistically anticipated frequency [32]. Numerous tumor-suppressor genes are silenced in various neoplasms in relation to aberrant promoter methylation, and within the absence of coding area mutations [32]. The underlying cause for the failure of cancer cells to sustain the CpG islands in an un-methylated condition remains unknown, posing a challenge for scientific investigation [32].

Recent research has also provided evidence that DNA methylation plays a crucial role in the control of non-CpG island promoters. For example, the expression of mammary serine protease inhibitor (MASPIN) in cancer cells, which lacks a CpG island in its promoter region, is regulated through DNA methylation [33]. A significant number of tissue-specific genes exhibit a correlation between gene activity and the presence of hypo methylation in their promoter regions. Furthermore, non-promoter CpG islands seem to be more susceptible to aberrant methylation than their adjacent promoter sequences; methylation can start in exonic regions and subsequently move to CpG islands in various locations, including promoter regions [34]. For instance, the degree of expression of the non-CpG island Oct-4 promoter is significantly impacted by methylation [34]. Aberrant methylation of these commonly unmethylated CpG islands is related to under-acetylated histones and a shift to a transcriptionally repressive chromatin structure [34].

Mechanisms of aberrant DNA methylation in human cancer

Epigenetic alterations, like DNA methylation, function as a secondary mechanism to silence TSGs in addition to genetic changes. The dysregulation of gene expression due to abnormal activity of these crucial epigenetic factors has been implicated in various types of cancers [34,35,36]. Aberrant DNA methylation in human carcinomas may result from the loss of a CpG island protection factor, mis-targeting of a DNMT complex, and alterations in replication timing. Although aberrant DNA methylation is a vast phenotype in most cancers, identifying the specific alterations riding the tumor phenotype can guide therapeutic strategy [36]. While the exact mechanisms behind the modification of the DNA methylation pattern remain unclear, available data indicate that DNMTs are responsible for the development of the DNA methylation pattern [36]. Consequently, the deregulation of DNMT expression may play a role in the overall alteration of DNA methylation patterns [36]. DNMT and demethylase (MBD2) are considered crucial factors in the abnormal DNA methylation observed in tumors. Elevated levels of DNMT and MBD2 expression could potentially advance tumor development through hypermethylation-induced silencing of TSGs in CpG islands. Recent reports have shown that aberrant methylation isn't confined to only a few genes or promoter areas and the majority of cancer cells exhibit abnormal patterns of DNA methylation, such as hypermethylation of gene promoter CpG islands and global demethylation of the genome, as seen in colon cancer. It also can result in the loss of genomic imprinting, which is an epigenetic phenomenon that occurs during the unequal allocation of chromosomes inherited from each parent during embryonic development [37,38,39]. A variety of genes have been demonstrated to be hypermethylated in colorectal tumors, including known TSGs [39] (Table 1). For example, the inactivation of the cyclin-dependent kinase inhibitor P16 through methylation can lead to the disruption of cell-cycle regulation, which may result in a growth advantage for the affected cells [40].

**Table 1:** Selected genes are affected by promoter methylation in normal colon mucosa and colorectal cancer. Source: [39]. NA, not applicable

Gene	Is it expressed in colon cancer?	Age-related methylation? (Quantitative)	Age-related methylation? (Sensitive)	Methylation in colon cancer (%)
PAX6	Yes	Yes	No	70
EGFR	Yes	Yes	No	0
P16	Yes	No	NA	20-30
MDR1	Yes	No	NA	20-30
IGF2	Yes	Yes	No	70
N33	Yes	Yes	No	80
RIZ1	No	No	NA	10-20
THBS1	Yes	No	NA	50
TIMP3	Yes	No	NA	27
HLTF	No	No	NA	50
CSPG2	Yes	Yes	No	70
CALCA	No	No	NA	50
P14/ARF	Yes	No	Yes	10-20
APC	Yes	No	NA	10-20
COX2	Yes	Yes	No	10-20

Hypomethylation has been implicated in benign hyperplasia [40], and various malignancies, such as in patients with immunodeficiency, centromeric instability, facial abnormalities, and other cancers where pericentric heterochromatin regions on chromosomes 1 and 16 are hypomethylated. It’s also clear that tumorigenesis may benefit from hypomethylation in both gene-coding and non-coding areas of the genome, leading to chromosomal instability (CIN) and the activation of proto-oncogenes such as cMYC and H-RAS [40,41]. It has been reported that hypomethylation of non-CpG island (non-CGI) promoters is less common than hypermethylation of CGI promoters, which can lead to the upregulation of oncogenes and proto-oncogenes. As tumor progression occurs, there is a greater loss of methylation, with metastatic lesions exhibiting more demethylation than primary tumors [41]. TSGs like p16, MLH1, and BRCA1 have also been shown to undergo tumor silencing through hypermethylation. These genes play critical roles in various cellular processes necessary for cancer progression, including DNA repair, cell cycle regulation, cell adhesion, apoptosis, and angiogenesis [41], which include APC, p53, p27, MSI, LOH 18q, deletion 5q allele, and DNA hypermethylation [38]. However, the combined effects of accumulated hypermethylation and hypomethylation on CRC development and outcome remain understudied [42,43].

Histone modifications 

Furthermore, post-translational covalent modifications at histone tails play a crucial role in controlling chromatin structure and gene expression [42,43]. Each of the enlarged globular histone proteins in a nucleosome contains a functional side chain or tail that is filled with essential lysine and arginine residues [43]. As a dynamic molecule with a couple of structures, there are two basic forms of chromatin: heterochromatin and euchromatin. In contrast to DNA methylation, histone modifications can cause either activation or repression, depending on the residues that are altered and the type of changes that are made [43,44]. At least seven distinct types of histone modifications (covalent modifications) exist, including acetylation, methylation, phosphorylation, ubiquitination, sumoylation, citrullination, and ADP-ribosylation [44]. Combinations of these alterations are believed to form the *histone code*, which is responsible for regulating chromatin conformation and gene expression levels. Acetylation and methylation, two of the seven modifications highlighted, are extensively studied and are known to play a significant role in CRC pathogenesis [44]. Among the recognized adjustments, acetylation has the greatest ability to unravel the chromatin since it neutralizes the fundamental rate of lysine [44]. In mammals, silent heterochromatin is associated with low levels of histone acetylation, whereas actively transcribed euchromatin usually contains high levels of histone acetylation [45].

There is a lot of crosstalk in the intricate relationship between chromatin shape, histone modification, and DNA methylation. In the epigenetic interaction between DNA methylation and histone modification, aberrant DNA methylation can affect chromatin structure and gene expression, whereas dysregulation of histones and associated adjusting proteins can also induce aberrant DNA methylation [46]. Histone modifications may be divided into two categories: maintaining global order and coordinating DNA-based biological processes. To create a global chromatin environment, modifications help to partition the genome into well-defined domains: euchromatin, where DNA is kept accessible for transcription, and heterochromatin, where chromatin is inaccessible for transcription [46,47]. Histone modification patterns are dynamically regulated by using enzymes that add and eliminate covalent changes to histone proteins. Histone acetylation involves the reversible alteration of lysine residues on histone tails and is regulated by histone acetyltransferases (HATs) [47]. Histone methyltransferases (HMTs) add acetyl and methyl groups, respectively, while histone deacetylases (HDACs) and histone demethylases (HDMs) remove acetyl and methyl groups, respectively [47]. HDACs and HATs served as transcriptional co-activators or co-repressors, separately. HDACs are regularly discovered overexpressed in different types of cancer. Among the various enzymes involved in histone modification, HDACs have been extensively investigated and are considered the most well-characterized proteins. Their pivotal roles in the development of CRC have been established through numerous studies [47,48]. HDACs can be categorized into three distinct families: class I, class II, and class III NAD-dependent enzymes of the Sir family [48]. In a specific subset of CRCs, there is an upregulation of various class I HDACs, such as HDAC1 in 36.4% of cases, HDAC2 in 57.9%, and HDAC3 in 72.9% of CRC specimens. Furthermore, worse patient survival in CRC has been linked to elevated HDAC expression levels. HDAC2 expression was detected in 81.9% of colorectal carcinoma, 62.1% of colorectal adenoma, and 53.1% of normal tissues [48].

Recent studies have shown that particular histone adjustments and chromatin conformation have appeared to act, in relation to DNA methylation, to control gene expression to intercede CRC pathogenesis. The histone tails are concerned with large covalent posttranslational modifications (PTMs) that cooperate to manipulate the chromatin state. Changes within the designs of histone PTMs have been broadly connected to cancer, both at the global level over the genome and at particular gene loci. Some PTMs can adjust the rate density among histones and DNA, impacting chromatin corporation and underlying transcriptional procedures; however, they can also serve as recognition modules for specific binding proteins that might also then signal for alterations in chromatin shape or function and apparently for cancer [48].

Genomic instability in CRC

CIN, MSI, and CIMP

**Figure 2:**
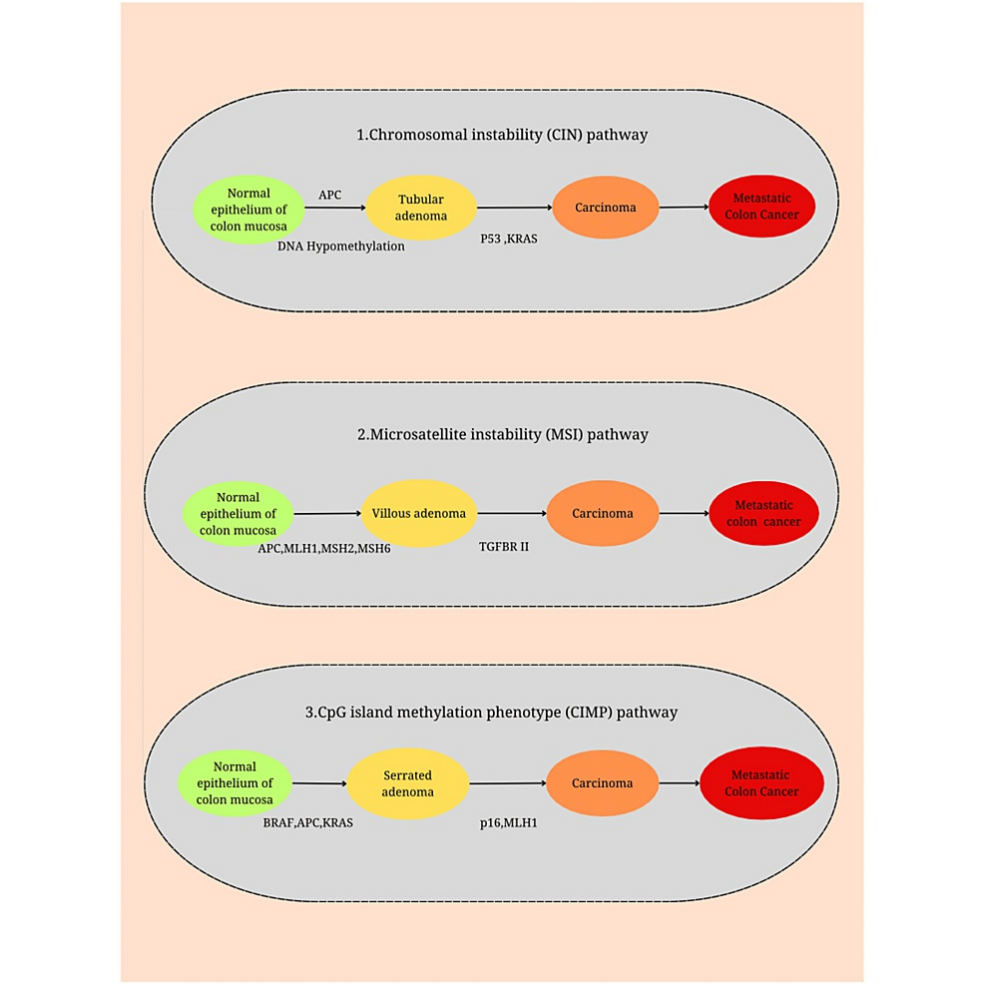
Genomic instability pathways in colorectal cancer. Original image depicted. Image credit: Asal Abolghasemi Fard.

It is considered that CRC is a heterogeneous malignancy with three distinctive, but overlapping, molecular phenotypes reflecting different forms of DNA instability [48,49] (Figure 2). The CIN pathway is considered the foremost common phenotype that represents 85% of tumors [5], and it is characterized by gross chromosomal lesions (gains and losses). However, the underlying mechanism stays uncertain [49]. The malignant cells in CIN tumors are frequently aneuploidy and uncover large-scale chromosomal rearrangements. Moreover, DNA aneuploidy, an established marker for CIN, is located in the majority of sporadic CRC and has been connected to poor prognosis. CIN is classically characterized as an increase in the rate at which numerical or structural chromosomal abnormalities are obtained in a cancer cell. In the context of identifying somatic mutations in genes associated with CIN in colorectal tumors, the utilization of a collection comprising 102 human homologs derived from 96 known genes is crucial [49]. Within a batch of 132 CRC cases, somatic mutations were identified in five genes [49]. Later, it was proven that these mutations cause CIN and abnormalities of chromatin cohesion in human cells. CIN results in the loss of the wild allele of TSGs like APC, P53, and SMAD4, which typically inhibit the development of malignant traits [49]. Recently, it has been shown that disruption of mitotic checkpoint assembly genes can also lead to CIN because checkpoint-defective cells can complete mitosis with inappropriately aligned chromosomes [50].

The second most common molecular phenotype is the microsatellite instability (MSI) pathway. MSI is diagnosed through somatic changes and is caused by different insufficiencies within the DNA mismatch-repair system, leading to a huge increase in the mutation rate [50]. MSI is detected in around 11% to 17% of all CRCs; 3% of them are related to Lynch syndrome (hereditary nonpolyposis CRC because of germline mutations in MMR proteins), and the others are a result of acquired hypermethylation of the promoter of the MLH1 gene [50]. Individuals with MSI are identified by the presence of common insertion and deletion mutations in repetitive DNA sequences. MSI plays a crucial role in the formation of colorectal tumors exhibiting a hypermutable phenotype. Due to their composition of extensively repetitive sequences, these regions exhibit a greater inclination toward acquiring mutations compared to other segments of the genome [50]. A distinct group of CRCs also exhibits a distinctive mutator phenotype, leading to MSI and mutations at various gene loci, including TGFbRII and BAX. This particular phenotype typically arises from the inactivation of mismatch repair (MMR) genes and other genes like MSH6 and PMS2, which are linked to the onset of Lynch syndrome, elevating the likelihood of cancer [51].

The last group is CIMP, which represents about 15% of CRCs [51,52], which was discussed before.

 DNMTs

The DNA methylation process in mammals is generated by DNMT enzymes, which transfer a methyl group to the S-adenosylmethionine to promote genome-extensive methylation patterns and gene silencing and also facilitate the catalytic addition of methyl groups to the 5th position of the cytosine rings of CpG dinucleotides. It contains DNMT1, DNMT3a, and DNMT3b, which transfer methyl groups from S-adenosylmethionine to DNA in either a de novo (DNMT3a and DNMT3b) or maintenance (DNMT1) route. It has been observed that overexpression of these genes can cause aberrant methylation of normally unmethylated CpG islands, similar to what is found in CRC [52].

DNMT1 is responsible for the maintenance of methylation and has a 30- to 40-fold preference for hemimethylated DNA. The most recent data confirmed that DNMT1 exhibits de novo DNMT activity [52]. The highest concentration of DNMT1 is found in cells, and it is typically transcribed during the S phase of the biological cycle, which is dependent on DNA replication. DNMT2 is characterized based on its resemblance to the bacterial-type cytosine Ⅱ-5 methyltransferase [52]. It is by far the most robustly conserved and widely distributed in mammals. On the other hand, recent research has demonstrated that, although lacking DNMT activity, DNMT2 can mediate tRNA methylation, indicating that there is still more to learn about it [52,53].

DNMT3s (DNMT3A and DNMT3B) play a key role in de novo methylation, being responsible for setting up the methylation patterns essential throughout embryonic improvement. It was observed that DNMT3A and DNMT3B act independently of DNA replication and display an identical preference for each unmethylated and hemimethylated DNA. Yet, the catalytic function of these particular enzymes remains unknown [53].

 De novo DNMTs 

The process of de novo methylation commonly begins at the outskirts of a promoter CpG island and advances toward the center of the island [53]. The term *de novo methylation *describes the methylation of DNA without the presence of a preexisting methylation pattern in the DNA template. Maintenance methylation, on the other hand, happens during DNA replication and consists of replicating the methylation pattern from the unreplicated DNA strand to the newly replicated strand. The de novo DNMTs are accountable for the establishment of gene methylation patterns during development, playing a pivotal role in gene regulation [53,54]. Through diverse mechanisms, CpG methylation can inhibit the binding of cis-binding factors like AP-2, CREB, E2F, CBF, and NF-KB, ultimately resulting in the transcriptional inactivation of genes. The exact mechanism underlying the loss of protection against abnormal DNA methylation in the majority of carcinomas, including CpG islands and nearby sequences, is still not fully comprehended [54]. Ongoing research suggests that certain sequences within the promoter region can serve as docking sites for repression complexes, which consist of HDACs, MBDs, DNMTs, and other proteins. As a result, the exonic sequences become hypermethylated, while their histones undergo hypoacetylation and methylation [54].

CGI 

CGIs are brief sequences that contain a high number of CpG dinucleotides and can be found within the 59 regions of around 50% of all human genes [54]. The loss of gene expression is linked to the methylation of cytosine within 59 CGIs, which has been observed in various physiological conditions including X chromosome inactivation and genomic imprinting. CGIs have been found to exhibit abnormal methylation patterns in various genetic disorders like Fragile-X syndrome, as well as in aging cells and neoplasia [54,55]. Therefore, it has been suggested that CGI methylation functions as an alternative means of gene inactivation in cancer. The lack of clear understanding regarding the causes and global patterns of CGI methylation in human cancers persists. Previous studies have suggested that aging might contribute to this phenomenon, as methylation of various CGIs has been observed in an age-related manner in both normal colon mucosa and CRC [55]. Furthermore, abnormal methylation of CGIs has been linked to the MSI phenotype in CRC, as well as exposure to specific carcinogens. However, the comprehension of abnormal methylation in CRC has been hindered to some extent due to the limited analysis of CGIs conducted so far. It has been observed that around half of the tumor-suppressor genes, which are known to be mutated in the germline of patients with familial cancer syndromes, also exhibit abnormal methylation in sporadic cancers. Notable examples of such genes include VHL, p16, and hMLH1. In cancer, the methylation of tumor-suppressor genes is commonly associated with the absence of gene transcription and the lack of coding region mutations. Consequently, it is hypothesized that CGI methylation acts as an alternative means of gene inactivation in cancer [55].

## Conclusions

In recent years, there has been a growing understanding of the relationship between cancer occurrence and advancement with epigenetic abnormalities. It is now known that disruptions in epigenetic processes can combine with mutations to trigger cancer, contributing to tumor growth. In the context of CRC research, significant emphasis has been placed on studying abnormal DNA methylation, particularly the occurrence of global hypomethylation and local hypermethylation in most cases. In the early stages of polyp-cancer progression, it is common to observe abnormal DNA methylation patterns that can impact the development of CRC. However, there are also changes in methylation that occur later in the sequence, which may influence the disease's progression. Many aspects of epigenetics remain unknown despite ongoing research. Researchers are continuously discovering new mechanisms and how they impact diseases, especially cancer. Understanding the cellular and molecular mechanisms driving the start and progression of tumorigenesis has created opportunities for the development of targeted and highly effective therapeutic interventions.
